
JOURNAL INFORMATION
==============================
NLM Title Abbreviation: ScientificWorldJournal
Journal ID: ScientificWorldJournal
NLM Journal ID: 101131163
Journal ID: TSWJ

The Scientific World Journal

ISSN: 2356-6140
EISSN: 1537-744X
Publisher: Wiley

ARTICLE INFORMATION
==============================
PMCID: PMC6084731
DOI: 10.1100/tsw.2001.417
Article ID: 753195
Article version: 1
Subjects: Extended Abstract

Exercise-Induced Calcitonin Gene-Related Peptide (CGRP) Increase during Normoxia and Hypoxia

Hasbak P. 1 2 3
Lundby C. 4
Olsen N. V. 4
Schifter S. 2
Kanstrup I. L. 1
1 Department of Clinical Physiology and Nuclear Medicine, University Hospital of Herlev, Herlev, Denmark
2 Department of Clinical Physiology and Nuclear Medicine, University Hospital of Glostrup, Glostrup, Denmark
3 Department of Clinical Experimental Research, University Hospital of Glostrup, Glostrup, Denmark
4 Department of Pharmacology, Panum Institute, University of Copenhagen, Denmark
Publication date: 2001
Electronic publication date: 2001 Feb 16
Volume: 1
First page: 41
Last page: 41
Copyright: Copyright © 2001 P. Hasbak et al.
Copyright year: 2001
License: This is an open access article distributed under the Creative Commons Attribution License, which permits unrestricted use, distribution, and reproduction in any medium, provided the original work is properly cited.
License URL: https://creativecommons.org/licenses/by/3.0/

==============================
4th International Meeting on Calcitonin Gene-Related Peptide (CGRP)
*TheScientificWorld* (2001) 1(S1), 41
ISSN 1532-2246; DOI 10.1100/tsw.2001.417

### EXERCISE-INDUCED CALCITONIN GENE-RELATED PEPTIDE (CGRP) INCREASE DURING NORMOXIA AND HYPOXIA

P. Hasbak[a,b,c], C. Lundby[d], N.V. Olsen[d], S. Schifter[b], and I.L. Kanstrup[a]
[a]Department of Clinical Physiology and Nuclear Medicine, University Hospital of Herlev, Herlev; [b]Department of Clinical Physiology and Nuclear Medicine, University Hospital of Glostrup, Glostrup; [c]Department of Clinical Experimental Research, University Hospital of Glostrup, Glostrup; [d]Department of Pharmacology, Panum Institute, University of Copenhagen, Denmark

To date calcitonin gene-related peptide (CGRP) is the most potent vasodilatatory peptide described. Evidence indicates that the level of CGRP increases during exercise in humans. Furthermore, hypoxia, lactic acid (LA) and sympathetic activity affect the release of CGRP. We therefore studied how CGRP levels would be influenced by maximal exercise in humans during normoxia, acute and prolonged hypoxia.

Twelve healthy volunteers were investigated at sea level (SL), day 1 (HA1) and day 5 (HA5) in high altitude (4559 m). CGRP, calcitonin (CT), noradrenalin (NA), adrenalin (A), LA, and heart rate (HR) were measured at rest and during maximal exercise ($W_{max}$) on a bicycle ergometer. At SL, HA1, and HA5, CGRP increased significantly from resting levels to $W_{max}$ along with NA, LA, HR, whereas A only increased significantly at sea-level and HA1. CT did not change at $W_{max}$. The CGRP levels at $W_{max}$ were significantly decreased at HA1 (74.3 ± 6.1 pmol/l; $p = 0.008$) and HA5 (69.6 ± 6; $p = 0.003$) compared to CGRP (80.9 ± 5.1 pmol/l) at SL. This pattern was similar for LA and HR at $W_{max}$ during acute and prolonged hypoxia, whereas the NA and A responses were not. At rest neither acute nor prolonged hypoxia changed the CGRP, CT, or LA levels, whereas NA, A, and HR changed significantly compared to SL.

The exact signal for release of CGRP cannot be stated from this study, but we hypothesize that the release of CGRP and LA are correlated and that CGRP does not solely act as a counter regulatory mechanism to the sympathetic vasoconstrictor NA.
